# Impact of a Large-Scale Handwashing Intervention on Reported Respiratory Illness: Findings from a Cluster-Randomized Controlled Trial

**DOI:** 10.4269/ajtmh.18-0644

**Published:** 2019-01-02

**Authors:** Nusrat Najnin, Karin Leder, Andrew Forbes, Leanne Unicomb, Peter J. Winch, Pavani K. Ram, Fosiul A. Nizame, Shaila Arman, Farzana Begum, Shwapon Biswas, Alejandro Cravioto, Stephen P. Luby

**Affiliations:** 1 International Centre for Diarrheal Disease Research, Bangladesh (icddr,b), Dhaka, Bangladesh;; 2 Department of Epidemiology and Preventive Medicine, School of Public Health and Preventive Medicine, Monash University, Melbourne, Australia;; 3 Johns Hopkins Bloomberg School of Public Health, Baltimore, Maryland;; 4 University at Buffalo, Buffalo, New York;; 5 Department of Medicine, Rangpur Medical College Hospital, Rangpur, Bangladesh;; 6 Facultad de Medicina, Universidad Nacional Autónoma de Mexico, Ciudad de Mexico, Mexico;; 7 Stanford University, Stanford, California

## Abstract

We assessed the impact of handwashing promotion on reported respiratory illness as a secondary outcome from among > 60,000 low-income households enrolled in a cluster-randomized trial conducted in Bangladesh. Ninety geographic clusters were randomly allocated into three groups: cholera-vaccine-only; vaccine-plus-behavior-change (handwashing promotion and drinking water chlorination); and control. Data on respiratory illness (fever plus either cough or nasal congestion or breathing difficulty within previous 2 days) and intervention uptake (presence of soap and water at handwashing station) were collected through monthly surveys conducted among a different subset of randomly selected households during the intervention period. We determined respiratory illness prevalence across groups and used log-binomial regression to examine the association between respiratory illness and presence of soap and water in the handwashing station. Results were adjusted for age, gender, wealth, and cluster-randomized design. The vaccine-plus-behavior-change group had more handwashing stations with soap and water present than controls (45% versus 25%; *P* < 0.001). Reported respiratory illness prevalence was similar across groups (vaccine-plus-behavior-change versus control: 2.8% versus 2.9%; 95% confidence interval [CI]: −0.008, 0.006; *P* = 0.6; cholera-vaccine-only versus control: 3.0% versus 2.9%; 95% CI: −0.006, 0.009; *P* = 0.4). Irrespective of intervention assignment, respiratory illness was lower among people who had soap and water present in the handwashing station than among those who did not (risk ratio_adjusted_: 0.82; 95% CI: 0.69–0.98). With modest uptake of the handwashing intervention, we found no impact of this large-scale intervention on respiratory illness. However, those who actually had a handwashing station with soap and water had less illness. This suggests improving the effectiveness of handwashing promotion in achieving sustained behavior change could result in health benefits.

## INTRODUCTION

Acute respiratory infections continue to be a major cause of mortality in low-income countries.^1,2^ Many respiratory infections are transmitted via infected droplets, but some viruses including the respiratory syncytial virus infecting the respiratory tract can also be spread from one person to another by hand contact.^3,4^ The focus of many hand hygiene interventions has been to reduce diarrhea, but data from a systematic review and a meta-analysis show that hygiene behavior change, including handwashing with soap has also been effective in reducing respiratory illness.^5,6^ The commonly used indicator to assess health impact of handwashing interventions in most of these studies is self-reported or caregiver-reported respiratory illness and, therefore, study findings may be subjected to reporting bias. Few studies have objectively measured the impact of handwashing on respiratory illness.^7,8^ For example, Cowling et al. objectively measured transmission of respiratory infection by using reverse-transcription polymerase chain reaction of nasal and throat swabs and reported that hand hygiene interventions prevented household transmission of influenza virus.^8^ Despite benefits for both diarrhea and respiratory infection prevention, hand hygiene practices (washing hands with soap) are suboptimal. A systematic review of 42 studies estimated that 19% of the world population washes hands with soap after contact with excreta.^9^ Structured observations of residents of rural Bangladesh found that only 1% of people washed their hands with soap before eating and before feeding a child and only 14% washed their hands with soap after defecation.^10^ Most previous efficacy studies reporting the impact of intense implementation of hygiene behavior change on respiratory illness have been small, involving up to 6,000 people.^5,6^ Upscaling known effective interventions is essential for improving global health^11^; however, the impact of implementing hygiene promotion programs on respiratory illness on a large scale is still unclear.^12,13^

Accurately assessing handwashing behaviors is problematic. Self-reported handwashing consistently overestimates observed behavior.^10,14,15^ Direct observation of handwashing by trained staff is both highly resource intensive and also biased, as the presence of an observer alters the handwashing behavior.^16,17^ Assessment of handwashing behavior through a low-cost proxy measure such as presence of soap and water in a designated handwashing station is a practical alternative and has been associated with lower rates of respiratory illness in some settings, but not in others.^18–21^

We conducted a cluster-randomized controlled trial in 2011–2013 among > 60,000 low-income households of metropolitan Dhaka, Bangladesh. The primary aim of the study was to evaluate the impact and feasibility of a mass cholera vaccination program in reducing diarrhea due to *Vibrio cholerae* in a high-incidence urban area. We have reported already that vaccination reduced the incidence of diarrhea attributable to *V. cholerae* in this community.^22^ This present article reports a prespecified secondary outcome of the trial to examine effects of an at-scale intervention under real-world conditions to promote handwashing with soap on reported respiratory illness. We hypothesized that scaling up a community-based handwashing intervention could reduce respiratory illness. We also examined whether the presence of soap and water at primary handwashing stations was associated with a reduction in respiratory illness, irrespective of intervention assignment of participants.

## METHODS

### Trial design and participant selection.

We conducted a cluster-randomized controlled trial in low-income communities of the Mirpur area of urban Dhaka. Details of the study methods including participant selection procedures have been published elsewhere.^22^ In short, the criteria that we used to select high-risk, cholera-prone study areas were low per capita income, poor sanitation, unsafe water use, sharing of water source, and poor living conditions. The study area was divided into 90 geographic clusters, with 30 m buffer zones around each cluster created to prevent contamination of the intervention across clusters. The selection criteria enabled having homogenous study participants across the clusters.

### Interventions.

#### Handwashing and water treatment promotion.

**Figure 1.**
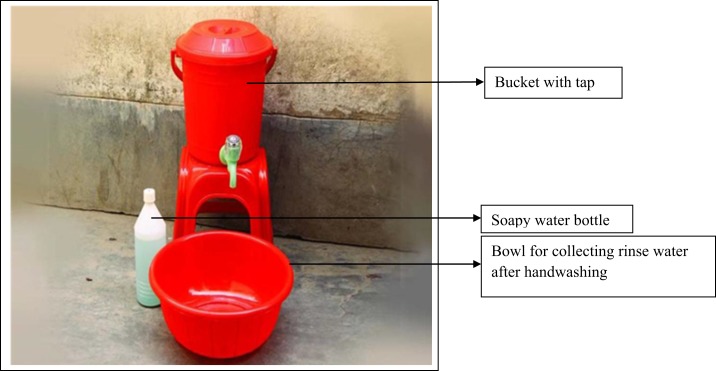
Handwashing station (includes bucket with tap, bowl, and soapy water bottle). This figure appears in color at www.ajtmh.org.

Handwashing and point-of-use water treatment promotion interventions both included hardware and behavior-change-communication activities and messages that were developed based on the integrated behavioral model for water sanitation and hygiene theoretical framework.^23,24^ Details about the interventions and how these were delivered in the study community have been reported elsewhere.^25^ In short, handwashing intervention hardware included a bucket with a tap, soapy water bottle,^26^ and a bowl to collect rinse water after washing hands (Figure 1).^25^ Soapy water was prepared by mixing a commercially available sachet of powdered detergent (∼US$ 0.03) with 1.5 L of water in a plastic bottle with a hole punched in the cap. The handwashing station hardware was provided free of charge to intervention compounds, but participating compounds had to supply either their own bar soap (∼US$ 0.35) or detergent sachets to make the soapy water. The behavior-change intervention also included point-of-use water treatment. The water treatment intervention hardware consisted of a dispenser containing liquid sodium hypochlorite.^25^ Study participants used their own water vessels for treating water.

A nongovernmental organization, Dushtha Shasthya Kendra (DSK), delivered the behavioral intervention through community health promoters. In the study area, several households often shared a common water source, kitchen, and toilets; therefore, we mostly provided the handwashing and water treatment intervention hardware at the compound level, although the behavior-change-communication messages were delivered both at compound and household levels. Within 3 months of cholera vaccination, the community health promoters visited each compound, discussed the trial, delivered the handwashing intervention, and specifically encouraged household members to wash their hands after defecation, after cleaning child’s anus, and before preparing food. The point-of-use intervention was rolled out 3 months later. During the initial 2 months after placement of each type of hardware, the promoters were instructed to visit each compound at least three times. After this period, the frequency of compound visits was reduced to twice monthly. The promoters also managed any problems related to intervention hardware.

#### Vaccine.

The cholera vaccine that was used in the study was ShanChol^™^ (Shantha Biotechnics-Sanofi, India), which is a killed whole cell, oral vaccine approved by the WHO as safe and effective against cholera.^27,28^ Details of vaccine transportation, storage, and administration to the study population have been previously reported.^22^

The study interventions that are not the focus of this article include point-of-use water treatment intervention and cholera vaccine. Details about these interventions including uptake have been described elsewhere.^22,25^

### Randomization and allocation concealment.

Ninety clusters were randomly allocated into three groups: 1) a cholera-vaccine-alone group (denoted as “vaccine-only” group hereafter), 2) a combined cholera-vaccine and behavior-change-communication intervention group (denoted as “vaccine-plus-behavior-change” group), and 3) a control group who continued regular habits and practices.^22^

Allocation concealment was not possible in this study because of the nature of interventions.

### Measurements.

The outcome of interest for this analysis was the prevalence of reported respiratory illness. During each month of the 2-year intervention period, data collectors visited a different set of 200 randomly selected study participants in the vaccine-plus-behavior-change group, and 100 participants in both the vaccine-only and control groups. They visited each of these households to collect information on respiratory illness, diarrhea, jaundice, and injuries within the 2 days before interview for each household member. These data collectors and the community health promoters from the DSK who delivered the behavior-change intervention products to the study participants worked independently of each other.

We classified people as having respiratory illness if they reported having fever plus either cough or nasal congestion or fever plus breathing difficulty.^12^ These unannounced home visits also assessed intervention uptake by observing the presence of soap/soapy water and water in the most convenient place for handwashing.

In an exploratory analysis, we compared the prevalence of respiratory illness among people who had soap/soapy water and water present in the primary handwashing station with those who did not, irrespective of intervention assignment.

During the study period through a separate six-monthly census survey, data collectors obtained information on births, deaths, and migrations of individuals from each house in the study area.^22^

### Study timeline.

**Figure 2.**
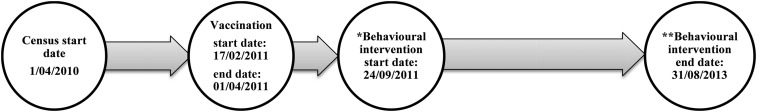
Study timeline. *For data analysis, we defined the behavioral intervention start date as September 24, 2011, which was the midpoint between the start and end dates of the handwashing intervention rollout. Data collection on respiratory illness and handwashing intervention uptake started from September 2011. **We ceased follow-up of the respiratory illness assessment at this time point.

For data analysis, we defined the behavioral intervention start date as September 24, 2011 (midpoint between the start and end dates of the handwashing intervention rollout). The behavior-change intervention and respiratory illness follow-up ceased on August 31, 2013 (Figure 2).

### Statistical methods.

We did not expect any direct association between cholera vaccine and respiratory illness. Therefore, respiratory illness prevalence in the vaccine-only group was expected to be similar to that in the control group. However, to preserve design-based scientific inference leveraging the randomized assignment of interventions (as prespecified before the trial), we chose to keep the vaccine-only group and the control group separate and compare them with vaccine-plus-behavior change group for our outcome of interest.

We compared baseline demographic characteristics of study participants across the three groups. The overall prevalence of respiratory illness across the follow-up period was calculated for each group, and we used binomial regression with a logarithmic link to calculate risk ratios (RR) directly and confidence intervals (CI) comparing groups, with robust standard errors to account for clustering.^29^ To examine the consistency of the intervention effects on the prevalence of reported respiratory illness over time, we divided the 2-year period of the intervention into quartiles (term 1 to term 4) and reported the prevalence for each quartile.

We performed an exploratory analysis to evaluate the relationship between presence of soap and water in the handwashing station and respiratory illness prevalence, regardless of the allocated intervention arm. We calculated respiratory illness prevalence according to the presence or absence of soap/soapy water and water in the primary handwashing station. We estimated RRs adjusting for age, and wealth of study participants, type of fuel used for cooking, and gender of respondents. We constructed a wealth index using principal component analysis.^30^ In the wealth index, we included household construction materials, education of respondents, and ownership of specific durable goods that are commonly used in Bangladesh and are considered to be discriminatory.^31^ We used the first factor from the principal component analysis, as this has been reported to best capture economic status.^32^ Based on their wealth score, we divided households into quintiles and adjusted for wealth quintile in the log-binomial regression models. Supplementary analyses adjusted for the first three principal components but results differed negligibly from using the first component only.

### Ethics.

Both verbal and written informed consent were obtained from each study participant before intervention and data collection started. Signature (or thumbprint, if illiterate) of the participants and parents/guardian of a child was obtained before their enrollment in the study. Informed written consent was again obtained from an adult study participant from each household before data were collected in each survey. The International Centre for Diarrheal Disease Research, Bangladesh ethics and research review committees approved the methods of consent gathering for this study. Data were kept anonymous throughout the study period and during analysis to maintain confidentiality. The study was registered at ClinicalTrials.gov (registration number: NCT01339845).

## RESULTS

**Figure 3.**
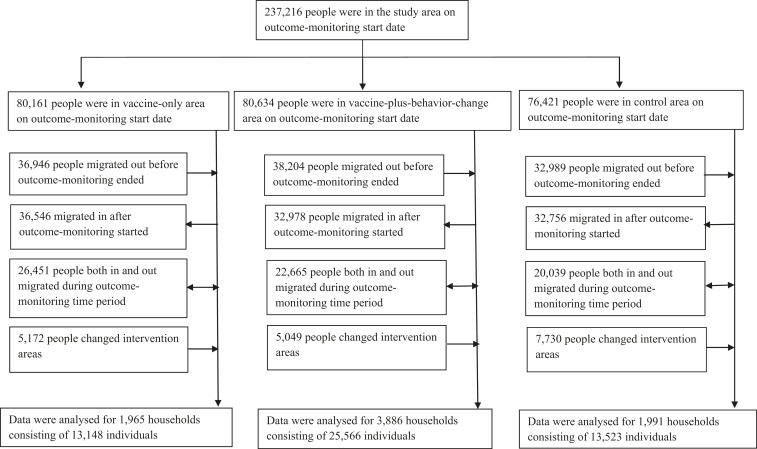
Participant flow during study period.

The census team identified a total of 237,216 people residing in the study area on the behavioral intervention start date. Among them, 80,161 were in the vaccine-only group, 80,634 were in the vaccine-plus-behavior-change group, and 76,421 were in the control group (Figure 3).^25^ For the monthly assessments, data collectors visited 7,842 households consisting of 52,237 people during the intervention period. Among these households, 1,965 (consisting of 13,148 individuals) were from the vaccine-only, 3,886 (consisting of 25,566 individuals) were from the vaccine-plus-behavior-change, and 1,991 (consisting of 13,523 individuals) were from the control group (Figure 3). Demographic characteristics were similar across all groups apart from educational status of respondents, presence of a sanitary latrine, and monthly income, which were slightly higher in the vaccine-plus-behavior-change group (Table 1). The pre-intervention period demographic characteristics were also similar across groups, suggesting homogenous distribution of study participants.^25^

**Table 1:** Demographic characteristics of individuals and households across the intervention groups during the study intervention period (September 2011–August 2013)*

Characteristics of individuals	Vaccine-only group (*n* = 13,148) %	Vaccine-plus-behavior-change group (*n* = 25,566) %	Control group (*n* = 13,523) %
Age (mean, SD) (years)	24 (15.9)	25 (15.9)	24 (16.0)
≤ 5	11	11	11
> 5 to 15	21	20	22
> 15 to 50	62	63	61
> 50	6	6	6

Characteristics of households	Vaccine-only group (*n* = 1,965) %	Vaccine-plus-behavior-change group (*n* = 3,886) %	Control group (*n* = 1,991) %
Gender of the respondent (female)	82	84	85
Educational status of respondent			
No formal education	37	34	38
Below primary	16	17	16
Primary and some secondary	45	47	45
Above secondary	1	2	1
Source of drinking water (WASA supply water)†	80	82	85
Toilet shared among families	91	90	90
House construction material			
Roof			
Corrugated iron	85	83	83
Brick/concrete	14	17	17
Bamboo/wood/other	1	0.2	0.2
Floor			
Brick/concrete	92	94	94
Bamboo/wood/mud/sand/other	8	6	6
Wall			
Brick/concrete	71	77	71
Bamboo/wood/corrugated iron/other	29	23	29
Type of fuel used for cooking			
Natural gas	72	84	77
Wood/husk/charcoal/kerosene	22	12	17
Other (electric heater)	6	4	5
Monthly income (median, interquartile range) (US$‡)	141 (97)	155 (90)	141 (90)

* Some categories do not sum to 100% because of rounding.

† Other sources of drinking water include well, tube well, bottled water, water vendor, and pond/canal/river.

‡ 1 USD = 77.6568 Bangladesh taka (average exchange rate during 2012).

### Intervention uptake.

Uptake of behavior-change interventions was modest as previously reported.^25^ In short, during the intervention period, interviewers identified the presence of soap/soapy water and water (either reserved in a container or available at the tap) at 45% (1,729/3,886) of primary handwashing stations in vaccine-plus-behavior-change group compounds, in 22% (438/1,965) of the vaccine-only group compounds, and in 28% (556/1,991) compounds of the control group.

### Prevalence of respiratory illness across intervention groups.

**Figure 4.**
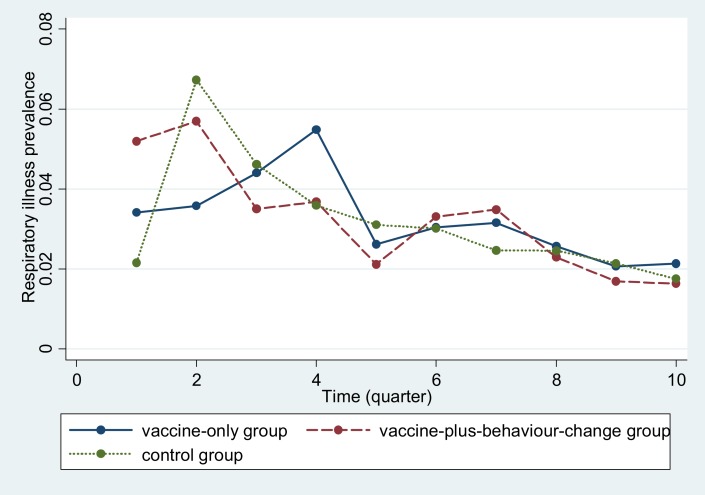
Reported respiratory illness prevalence within last 2 days across the groups during the intervention period (September 2011–August 2013). *Intervention time period (presented in quarters) started from quarter 2. This figure appears in color at www.ajtmh.org.

The overall reported respiratory illness prevalence (all intervention and age groups combined) within the last 2 days of interview was 2.9% (1,494/52,237 surveyed individuals). Respiratory illness prevalence was similar across the groups (vaccine-plus-behavior change versus control: 2.8% [708/25,566] versus 2.9% [388/13,523], 95% CI: −0.008, 0.006; *P* = 0.6; vaccine-only versus control: 3.0% [398/13,148] versus 2.9%; 95% CI: −0.006, 0.009; *P* = 0.4). On univariate regression analysis (adjusted for the cluster design), the prevalence of respiratory illness in the intervention groups was similar to that in the control group (vaccine-plus-behavior-change versus control: RR: 0.97; 95% CI: 0.76, 1.22; vaccine-only versus control: RR: 1.06; 95% CI: 0.82, 1.35). The results remained unchanged after adjusting these for age and wealth of study participants, and gender of respondent (data not shown). Even though the reported respiratory illness prevalence decreased in all groups over time, there was no difference in illness prevalence across intervention and control groups during the intervention period (Figure 4).

Children ≤ 5 years of age had the highest respiratory illness prevalence compared with children of other age groups. Even though reported respiratory illness among children ≤ 5 years was comparatively lower in the vaccine-plus-behavior-change group compared with other groups (Table 2), the difference was not statistically significant (vaccine-plus-behavior-change group versus control: 6.7% versus 7.4%; 95% CI: −0.03, 0.02; *P* = 0.4 and vaccine-only group versus control group: 7.1% versus 7.4%; 95% CI: −0.03, 0.03; *P* = 0.7).

**Table 2:** Reported respiratory illness prevalence within last 2 days of interview according to age and intervention groups during intervention period (September 2011–August 2013)

Age	All intervention groups combined (*N* = 52,237) %	Vaccine-only group (*n* = 13,148) %	Vaccine-plus-behavior-change group (*n* = 25,566) %	Control group (*n* = 13,523) %
< 5 years	7.0	7.1	6.7	7.4
> 5 to ≤ 15 years	1.9	2.1	1.7	2.0
> 15 to ≤ 50 years	2.5	2.6	2.5	2.4
> 50 years	2.6	2.8	2.6	2.3
All age groups combined	2.9	3.0	2.8	2.9

### Presence of soap/soapy water and water in handwashing station and prevalence of respiratory disease.

Overall (all groups combined), 35% (2,723/7,842) of the households had either soap or soapy water with water present in the primary handwashing station. People who had soap/soapy water and water present in the handwashing station reported lower respiratory illness prevalence (2.4% versus 3.0%, *P* < 0.001; RR_unadjusted_ = 0.80, 95% CI: 0.67, 0.95). The prevalence of having respiratory illness was approximately 18% less among people who had soap/soapy water and water present in handwashing station after adjusting for possible confounders (age and wealth of study participant, type of fuel used for cooking, gender of respondent, and cluster-randomized design of the trial): (RR_adjusted_: 0.82, 95% CI: 0.69, 0.98). The association of the presence of soap/soapy water plus water and respiratory illness did not vary by age.

## DISCUSSION

This study presents the impact of a large-scale community-based handwashing intervention trial on respiratory illness. We found no impact of the handwashing intervention on overall or age-specific reported respiratory illnesses. However, people who had soap/soapy water plus water present at their handwashing station, irrespective of intervention allocation, had lower prevalence of respiratory illness.

There are two potential explanations for the lack of impact of the handwashing intervention in this large-scale trial. First, it is possible that study participants followed the hand hygiene recommendations but that washing hands with soap does not reduce the burden of respiratory ilness in these communities. However, evidence from a systematic review and from a meta-analysis of small-scale efficacy studies suggests that washing hands with soap can effectively reduce respiratory illness in similar contexts.^5,6^ An alternate and more likely explanation is that there was insufficient uptake of the recommended handwashing behavior to interrupt respiratory pathogen transmission. This explanation is supported by the observation that people who actually had soap and water present at their handwashing station, regardless of intervention assignment, had lower respiratory illness prevalence. Our findings suggest that even though handwashing can effectively reduce respiratory illness in this context, in this large-scale trial, the intervention did not improve handwashing behavior sufficiently to measurably impact on respiratory illness.

The indicator of uptake for handwashing behavior in our study, namely, the presence of soap/soapy water plus water in the primary handwashing station, was 17% higher (45% versus 28%) in the vaccine-plus-behavior-change intervention group compared with the control group. Even though this increase seems low compared with some efficacy studies with more intense promotion of handwashing behavior,^33,34^ the handwashing intervention uptake was not much higher in our study compared with those of other large-scale interventions.^12,13^ For comparison, a project, Sanitation, Hygiene Education, and Water Supply in Bangladesh (SHEWA-B), aimed to improve hygiene, sanitation, and water supply for 20 million people in rural Bangladesh.^12^ During the first 2 years of the intervention period, the focus was to improve water sanitation and hygiene behavior through interpersonal communication and group discussions. By the end of this 2 years intervention period, the presence of water, soap, or ash in convenient handwashing location had increased up to 16% from baseline (baseline 47% versus postintervention 63%).^35^ Similarly, the national handwashing promotion program in Peru, targeting ∼28 million people, found no effect of a mass media intervention on handwashing behavior and combined the mass media campaign, although with more intense training and promotional activities at the community level increased the share of households with handwashing facilities by 4.9%.^13^ Neither SHEWA-B nor the Peru national handwashing program resulted in a measurable reduction in childhood diarrhea or respiratory illness.^12,13^ However, both SHEWA-B and the Peru national handwashing program were externally funded programmatic interventions targeting millions of people compared with our trial focused in one neighborhood of a large city. The reasons for poor uptake of this pretested intervention will be assessed and reported separately, but maintenance and management difficulties related to provision of shared handwashing facilities in intervention compounds may have contributed.

It is possible that the high-population migration rate in this study reduced the impact of the behavior-change intervention and so prevented an observable impact on respiratory illness risk. We have previously reported that large numbers of study participants moved outside the study area within the 2-year study period, and this might have limited the consistency of participants’ exposure to the hygiene behavior intervention.^25^ Uptake of the intervention was marginally (∼4%) higher among people who stayed in the study area for at least 1 year after the intervention started compared with those who migrated in or out.^25^ However, among people whose respiratory outcome was analyzed, we do not know how many were recent immigrants into the study areas and so could not directly explore the relationship between migration and respiratory illness.

In our study, the households that had soap and water present in the handwashing station irrespective of intervention assignment experienced less respiratory infection. The presence of soap and water in the handwashing station does not necessarily ensure that participants actually washed their hands or used soap. However, evidence suggests that people are more likely to wash their hands at key times if they have soap and water present in the handwashing station.^10,36^ An association between this surrogate measure of handwashing behavior and interruption in disease transmission has been observed in other studies that showed fewer child respiratory infections among participants with access to water for washing hands in the house.^18,19^ This protective effect of the presence of soap/soapy water and water in handwashing stations on respiratory illness that we observed in this study was for the overall study population rather than for any specific age group. Because these handwashing indicators are common among households with higher socioeconomic status^18^ and women in this context have been observed to practice better respiratory hygiene compared with men,^37^ we adjusted the results for both wealth and gender; the results remained significant. However, it was not possible to adjust for unmeasured confounders, such as intervention families taking more care to maintain a handwashing facility or providing better care for their children. In addition, one of the pathways that handwashing interventions may reduce the risk of respiratory disease is by preventing diarrhea that predisposes to subsequent respiratory infection.^38,39^ Because the intervention did not substantially impact diarrhea-related hospitalization rates by study groups,^25^ this complementary pathway to reduce respiratory infections was less likely to be active.

Our study has several limitations. The focus of the behavioral messages for washing hands was related mainly to defecation and food preparation events, as the goal of the main study was aimed at reducing diarrhea in the community rather than respiratory diseases. Even though hands have a potential role in transmission of respiratory viruses,^40,41^ focused behavioral interventions targeting reducing transmission of respiratory pathogens might be more effective in reducing illness prevalence. In fact, respiratory hygiene is often poorly practiced in low- and middle-income Bangladesh communities.^37^ A study conducted in Bangladesh reported that in 81% of the observed events, the participants coughed or sneezed into air (i.e., uncovered), and in 11% into their hands. No one washed their hands after coughing or sneezing into their hands.^37^ Another limitation is that it is possible that the intervention impacted on severe respiratory illness such as pneumonia but not on milder forms of self-reported respiratory symptoms at the community level that we assessed. Because severe respiratory infections represent the greatest public health burden, future evaluations would ideally assess this outcome.

Although the association of having soap and water present in the handwashing station and lower respiratory infection suggests that continued effort to develop low-cost strategies to improve population handwashing practices has the potential to improve child health, the interventions deployed in this trial did not impact respiratory illness. Changing handwashing behavior among large populations remains difficult, and so, such efforts should be rigorously evaluated so that the global community can learn from ongoing efforts and attempt to develop and optimize sound strategies.
